# Crystal structure and Hirshfeld-surface analysis of a monoclinic polymorph of 2-amino-5-chloro­benzo­phenone oxime at 90 K

**DOI:** 10.1107/S2056989023004668

**Published:** 2023-06-06

**Authors:** Doreswamy Geetha, Channappa N. Kavitha, Thayamma R. Divakara, Yeriyur B. Basavaraju, Hemmige S. Yathirajan, Sean Parkin

**Affiliations:** a Department of Studies in Chemistry, University of Mysore, Manasagangotri, Mysuru-570 006, India; b Department of Chemistry, Government Science College, Hassan-573 201, India; c T. John Institute of Technology, Bengaluru-560 083, India; d Department of Chemistry, University of Kentucky, Lexington, KY, 40506-0055, USA; University of Neuchâtel, Switzerland

**Keywords:** crystal structure, benzo­phenone oxime, polymorph, Hirshfeld surface

## Abstract

The synthesis and crystal structure of a monoclinic polymorph of 2-amino-5-chloro­benzo­phenone oxime, C_13_H_11_ClN_2_O, are presented. The mol­ecular conformation results from twisting of the phenyl and 2-amino-5-chloro benzene rings attached to the oxime group, which subtend a dihedral angle of 80.53 (4)°. In the crystal, centrosymmetric dimers are formed as a result of pairs of strong O—H⋯N hydrogen bonds. A comparison is made to a previously known triclinic polymorph, including differences in atom–atom contacts obtained *via* a Hirshfeld-surface analysis.

## Abstract 2

The synthesis and crystal structure of a monoclinic polymorph of 2-amino-5-chloro­benzo­phenone oxime, C_13_H_11_ClN_2_O, are presented along with a comparison to a previously determined triclinic form.

## 1. Chemical context

2-Amino-5-chloro­benzo­phenone is an ecologically friendly cross-linking agent. Benzo­phenone and related compounds have been reported to act as anti-allergic, anti-inflammatory, anti-asthmatic, and anti-anaphylactic agents (Evans *et al.*, 1987 ▸; Wiesner *et al.*, 2002 ▸; Sieron *et al.*, 2004 ▸). Benzo­phenone derivatives are widely used in sunscreen lotions, offering UV-A and UV-B protection (Deleu *et al.*, 1992 ▸). 2-Amino-5-chloro­benzo­phenone is used to produce inter­mediates for the synthesis of oxazolam drugs and inter­mediates for psychotherapeutic agents, such as chloro­diazepoxide and diazepam (Sternbach & Reeder, 1961*a* ▸,*b* ▸). 2-Amino­benzo­phenone and its derivatives have importance because of their applications in heterocyclic synthesis and medicines (Walsh, 1980 ▸) and are also used as anti-mitotic agents (Liou *et al.*, 2002 ▸). The growth and characterization of 2-amino-5-chloro­benzo­phenone single crystals was reported by Mohamed *et al.* (2007 ▸). Synthesis, herbicidal evaluation and structure–activity relationships of some benzo­phenone oxime ether derivatives was reported by Ma *et al.* (2015 ▸). The synthesis, physicochemical, and bio­logical evaluation of 2-amino-5-chloro­benzo­phenone derivatives as potent skeletal muscle relaxants was reported by Singh *et al.* (2015 ▸). Details of synthetic methodologies and the pharmacological significance of 2-amino­benzo­phenones as versatile building blocks was published by Chaudhary *et al.* (2018 ▸). The reactivity of oximes for diverse methodologies and synthetic applications was recently reported by Rykaczewski *et al.* (2022 ▸). In view of the general importance of benzo­phenone derivatives and those of 2-amino-5-chloro­benzo­phenone in particular, this paper reports the 90 K crystal structure and Hirshfeld-surface studies of a monoclinic form of 2-amino-5-chloro­benzo­phenone oxime, C_13_H_11_ClN_2_O, *mon*-2A-5CBO. A triclinic polymorph was recently published as a CSD communication (refcode REZSIB) by Lanzilotto, Housecroft *et al.* (2018 ▸). Some comparisons between the two crystal structures are presented. 
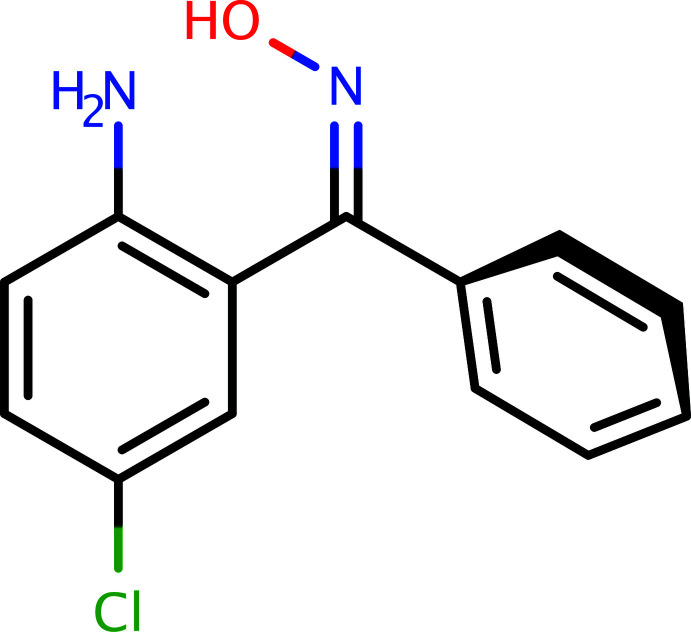


## 2. Structural commentary

**Figure 1:**
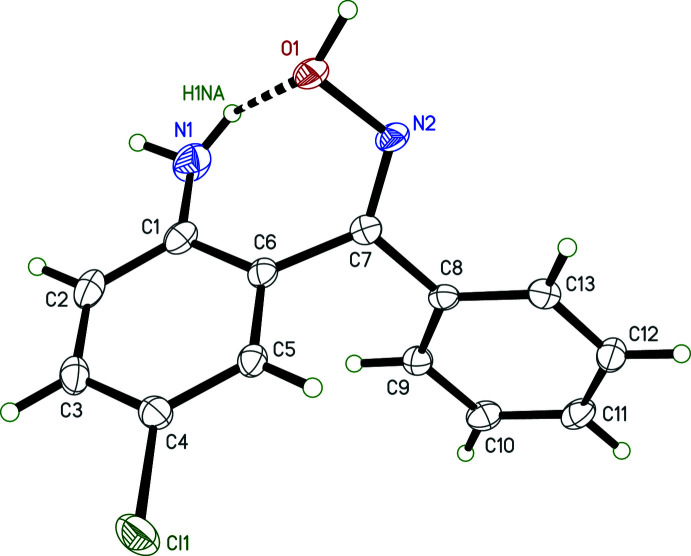
An ellipsoid plot (50% probability) of *mon*-2A-5CBO. The intra­molecular N—H⋯O hydrogen bond is shown as a dashed line.

**Figure 2:**
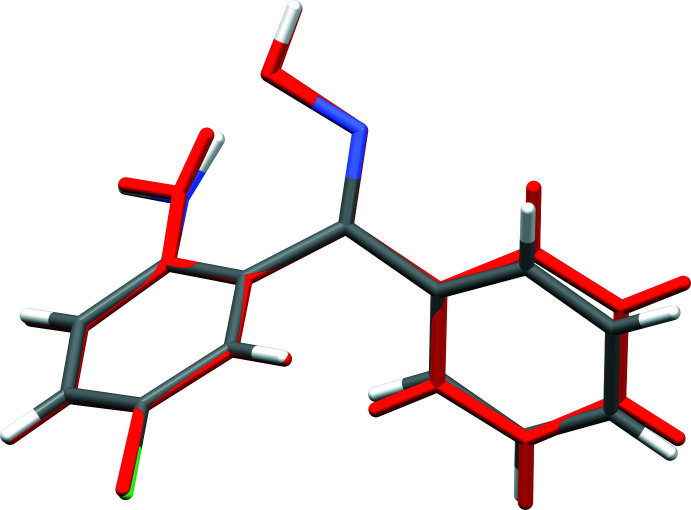
A least-squares fit overlay of *mon*-2A-5CBO and the triclinic polymorph REZSIB (red).

The overall conformation of the *mon*-2A-5CBO mol­ecule (Fig. 1 ▸) is determined by torsion angles about the C6—C7 and C7—C8 bonds that connect the chloro­aniline and phenyl rings to the oxime carbon, C7. These are held in check by an intra-mol­ecular hydrogen bond, N1—H1*NA*⋯O1 [*d_D⋯A_* = 2.8875 (19) Å, Table 1 ▸]. These torsion angles result in a dihedral angle between the two rings of 80.53 (4)°. The conformation defining torsion and dihedral angles are gathered in Table 2 ▸ along with those of the triclinic polymorph, REZSIB (Lanzilotto, Housecroft *et al.*, 2018 ▸). The conformations of the 2A-5CBO mol­ecules in the two polymorphs are quite similar, as shown by the overlay plot in Fig. 2 ▸. The r.m.s. deviation obtained from a weighted least-squares fit of all non-hydrogen atoms using OFIT in *SHELXTL-XP* (Sheldrick, 2008 ▸) is only 0.1315 Å, with the largest deviation being 0.267 Å for C12.

## 3. Supra­molecular features

**Figure 3:**
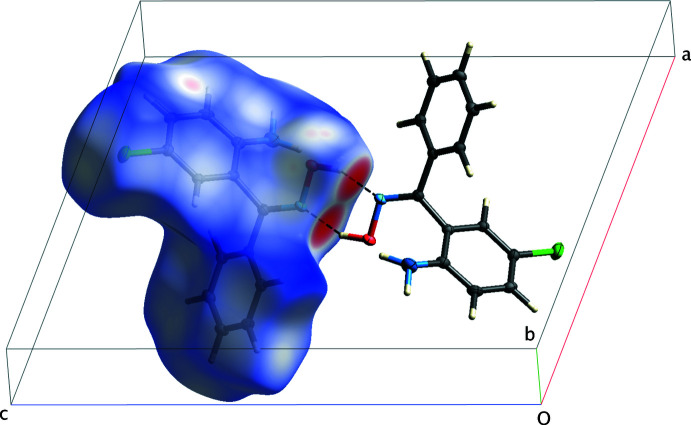
A partial packing plot of *mon*-2A-5CBO viewed approximately down the *b*-axis showing the Hirshfeld surface (left) and the 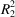 (6) centrosymmetric dimer formed by pairs of O—H⋯N hydrogen bonds (dashed lines and prominent red spots).

**Figure 4:**
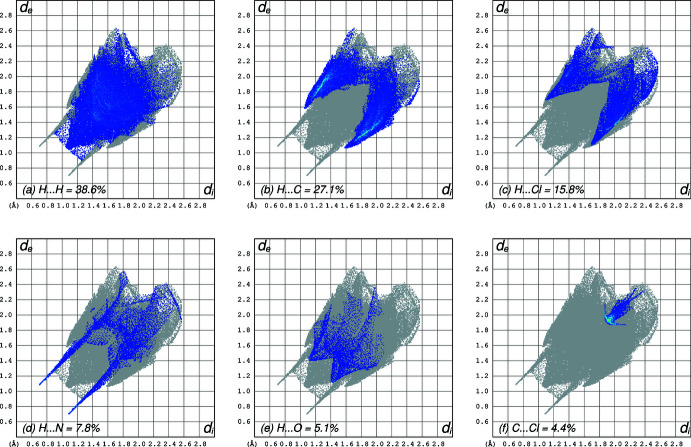
Hirshfeld surface two-dimensional fingerprint plots of *mon*-2A-5CBO showing (*a*) H⋯H, (*b*) H⋯C, (*c*) H⋯Cl, (*d*) H⋯N, (*e*) H⋯O, and (*f*) C⋯Cl close contacts.

The main supra­molecular constructs in the *mon*-2A-5CBO crystal structure are 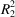 (6) centrosymmetric dimers that result from pairs (O1—H1*O*⋯N2^inv^ and O1^inv^—H1*O* ^inv^⋯N2, inv = 1 − *x*, 1 − *y*, 1 − *z*) of strong hydrogen bonds [*d_D⋯A_* = 2.7411 (16) Å, Table 1 ▸]. These are shown as dashed lines in Fig. 3 ▸ along with a representation of the Hirshfeld surface, as generated by *CrystalExplorer* (Spackman *et al.*, 2021 ▸), on which the hydrogen bonds are responsible for the prominent red spots. Similar dimer motifs are present in REZSIB. The most striking difference in packing between the two polymorphs is that REZSIB exhibits slip-stacked π–π overlap [inter­planar separation = 3.340 (2) Å, centroid–centroid distance = 3.897 (2) Å] of inversion-related (1 − *x*, −*y*, 1 − *z*) chloro­aniline rings, whereas *mon*-2A-5CBO does not. Hirshfeld surface 2D-fingerprint plots for *mon*-2A-5CBO are shown in Fig. 4 ▸ and the differences in contacts between the polymorphs are summarized in Table 3 ▸.

## 4. Database survey

A survey of the Cambridge Structural Database (CSD: v5.43 including all updates through November 2022; Groom *et al.*, 2016 ▸) returned 5507 hits for a search fragment consisting of unsubstituted benzo­phenone. A search using benzo­phenone oxime as the probe, however, returned only 35 entries. Of these, ten have a nitro­gen-bound functional group at the *ortho*-position of one of the benzene rings, while six have ‘any halogen’ attached at one of the *meta*-positions. In only two structures is this halogen a chlorine atom: YIFCIC (Lanzilotto, Prescimone *et al.*, 2018 ▸), C_15_H_11_Cl_2_FN_2_O_2_, systematic name 2-chloro-*N*-{4-chloro-2-[(2-fluoro­phen­yl)(hy­droxy­imino)­meth­yl]phen­yl}acetamide and REZSIB (Lanzilotto, Housecroft *et al.*, 2018 ▸), the triclinic (*P* 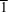 ) polymorph of the monoclinic (*P*2_1_/*n*) 2A-5CBO crystal structure described herein.

Some other related crystal structures include 2-amino-5-chloro­benzo­phenone as monoclinic (NUVFAL; Vasco-Mendez *et al.*, 1996 ▸) and triclinic (NUVFAL02; Javed *et al.*, 2018 ▸) polymorphs, benzo­phenone oxime (XULKUK; Sharutin *et al.*, 2002 ▸), and 2-benzo­yloxy-5-methyl­benzo­phenone (OCAMOV; Sieron *et al.*, 2004 ▸).

## 5. Synthesis and crystallization

**Figure 5:**
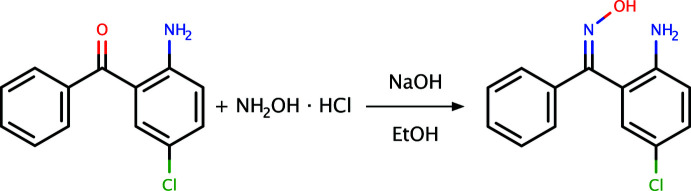
A general reaction scheme for the formation of 2A-5CBO.

The synthesis of 2A-5CBO (Fig. 5 ▸) was by a modification of Beckmann’s conversion of benzo­phenone to benzo­phenone oxime (Beckmann, 1886 ▸). In a 100 ml round-bottom flask fitted with a magnetic stirrer was placed a mixture of 100 mmol (23.2 g) of 2-amino-5-chloro­benzo­phenone, 120 mmol (7 g) of hydroxyl­amine hydro­chloride in 10 ml of ethanol. To this stirred mixture, 0.5 g of sodium hydroxide pellets was added in small portions. When the reaction became vigorous, the flask was placed in an ice bath. A condenser was attached to the flask and the mixture was refluxed for 5 minutes on a steam bath. The solution was cooled and poured into a beaker containing 5 ml of hydro­chloric acid and crushed ice. This was stirred until a precipitate formed. After filtering the precipitate with suction and washing with cold distilled water, the product was spread out on filter paper and air dried. The yield was 87%. X-ray quality crystals were obtained from methanol by slow evaporation (m.p.: 390–393 K).

## 6. Refinement

Crystal data, data collection and structure refinement details are summarized in Table 4 ▸. All hydrogen atoms were found in difference-Fourier maps. Those bound to carbon were subsequently included in the refinement using a riding model, with constrained distances fixed at 0.95 Å and *U*_iso_(H) values set to 1.2*U*_eq_ of the attached atom. The amine and oxime hydrogen atoms were refined freely.

## Supplementary Material

Crystal structure: contains datablock(s) I, global. DOI: 10.1107/S2056989023004668/tx2069sup1.cif

Structure factors: contains datablock(s) I. DOI: 10.1107/S2056989023004668/tx2069Isup2.hkl

Supporting information file. DOI: 10.1107/S2056989023004668/tx2069Isup3.cml

CCDC reference: 2265648

Additional supporting information:  crystallographic information; 3D view; checkCIF report

## Figures and Tables

**Table 1:** Hydrogen-bond geometry (Å, °)

*D*—H⋯*A*	*D*—H	H⋯*A*	*D*⋯*A*	*D*—H⋯*A*
N1—H1*NA*⋯O1	0.91 (2)	2.23 (2)	2.8875 (19)	128.7 (16)
O1—H1*O*⋯N2^i^	0.95 (2)	1.84 (2)	2.7411 (16)	156.4 (19)

Symmetry code: (i) 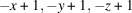 .

**Table 2:** Comparison of conformation-defining torsion and dihedral angles (°) in *mon*-2A-5CBO and CSD entry REZSIB

	*mon*-2A-5CBO	REZSIB^*a*,*b*^
Torsion angle		
N1—C1—C6—C7	−1.3 (2)	−5.6
C1—C6—C7—N2	60.8 (2)	56.7
C6—C7—N2—O1	0.5 (2)	7.8
Dihedral angle		
C1–C6/C8–C13	80.53 (4)	75.82

Notes: (*a*) The numbering scheme in REZSIB is different from *mon*-2A-5CBO; (*b*) Values from *Mercury* (Macrae *et al.*, 2020 ▸), therefore there are no SUs.

**Table 3:** Atom–atom contact coverages (%) for polymorphs *mon*-2A-5CBO and REZSIB

Atom contacts^*a*^	*mon*-2A-5CBO	REZSIB
H⋯H	38.6	43.6
H⋯C	27.1	17.6
H⋯Cl	15.8	13.6
H⋯N	7.8	8.8
H⋯O	5.1	6.4
C⋯Cl	4.4	6.0
C⋯C	0.0	3.9

Note: (*a*) Includes reciprocal contacts. All other contact percentages are negligible.

**Table 4:** Experimental details

Crystal data	
Chemical formula	C_13_H_11_ClN_2_O
*M*_r_	246.69
Crystal system, space group	Monoclinic, *P*2_1_/*n*
Temperature (K)	90
*a*, *b*, *c* (Å)	12.8264 (3), 5.5423 (1), 17.4082 (4)
β (°)	109.522 (1)
*V* (Å^3^)	1166.37 (4)
*Z*	4
Radiation type	Mo *K*α
μ (mm^−1^)	0.31
Crystal size (mm)	0.30 × 0.24 × 0.02

Data collection	
Diffractometer	Bruker D8 Venture dual source
Absorption correction	Multi-scan (*SADABS*; Krause *et al.*, 2015 ▸)
*T*_min_, *T*_max_	0.924, 0.971
No. of measured, independent and observed [*I* > 2σ(*I*)] reflections	26003, 2680, 2294
*R*_int_	0.042
(sin θ/λ)_max_ (Å^−1^)	0.650

Refinement	
*R*[*F* ^2^ > 2σ(*F* ^2^)], *wR*(*F* ^2^), *S*	0.033, 0.083, 1.05
No. of reflections	2680
No. of parameters	166
H-atom treatment	H atoms treated by a mixture of independent and constrained refinement
Δρ_max_, Δρ_min_ (e Å^−3^)	0.32, −0.27

Computer programs: *APEX3* (Bruker, 2016 ▸), *SHELXT* (Sheldrick, 2015*a* ▸), *SHELXL2019/2* (Sheldrick, 2015*b* ▸), *XP* in *SHELXTL* (Sheldrick, 2008 ▸), *SHELX* (Sheldrick, 2008 ▸) and *publCIF* (Westrip, 2010 ▸)’.

## supplementary crystallographic information

### Crystal data

**Table:**

C_13_H_11_ClN_2_O	*F*(000) = 512
*M**_r_* = 246.69	*D*_x_ = 1.405 Mg m^−^^3^
Monoclinic, *P*2_1_/*n*	Mo *K*α radiation, λ = 0.71073 Å
*a* = 12.8264 (3) Å	Cell parameters from 9975 reflections
*b* = 5.5423 (1) Å	θ = 2.5–27.5°
*c* = 17.4082 (4) Å	µ = 0.31 mm^−^^1^
β = 109.522 (1)°	*T* = 90 K
*V* = 1166.37 (4) Å^3^	Semi-regular block, pale yellow
*Z* = 4	0.30 × 0.24 × 0.02 mm

### Data collection

**Table:**

Bruker D8 Venture dual source diffractometer	2680 independent reflections
Radiation source: microsource	2294 reflections with *I* > 2σ(*I*)
Detector resolution: 7.41 pixels mm^-1^	*R*_int_ = 0.042
φ and ω scans	θ_max_ = 27.5°, θ_min_ = 2.4°
Absorption correction: multi-scan (*SADABS*; Krause *et al.*, 2015)	*h* = −16→16
*T*_min_ = 0.924, *T*_max_ = 0.971	*k* = −7→7
26003 measured reflections	*l* = −22→22

### Refinement

**Table:**

Refinement on *F*^2^	Primary atom site location: structure-invariant direct methods
Least-squares matrix: full	Secondary atom site location: difference Fourier map
*R*[*F*^2^ > 2σ(*F*^2^)] = 0.033	Hydrogen site location: mixed
*wR*(*F*^2^) = 0.083	H atoms treated by a mixture of independent and constrained refinement
*S* = 1.05	*w* = 1/[σ^2^(*F*_o_^2^) + (0.0334*P*)^2^ + 0.6832*P*] where *P* = (*F*_o_^2^ + 2*F*_c_^2^)/3
2680 reflections	(Δ/σ)_max_ = 0.001
166 parameters	Δρ_max_ = 0.32 e Å^−^^3^
0 restraints	Δρ_min_ = −0.27 e Å^−^^3^

### Special details

**Table:**

Experimental. The crystal was mounted using polyisobutene oil on the tip of a fine glass fibre, which was fastened in a copper mounting pin with electrical solder. It was placed directly into the cold gas stream of a liquid-nitrogen based cryostat (Hope, 1994; Parkin & Hope, 1998). Diffraction data were collected with the crystal at 90K, which is standard practice in this laboratory for the majority of flash-cooled crystals.
Geometry. All esds (except the esd in the dihedral angle between two l.s. planes) are estimated using the full covariance matrix. The cell esds are taken into account individually in the estimation of esds in distances, angles and torsion angles; correlations between esds in cell parameters are only used when they are defined by crystal symmetry. An approximate (isotropic) treatment of cell esds is used for estimating esds involving l.s. planes.

### Fractional atomic coordinates and isotropic or equivalent isotropic displacement parameters (Å^2^)

**Table:**

	*x*	*y*	*z*	*U*_iso_*/*U*_eq_	
Cl1	0.63274 (3)	0.54249 (7)	0.94134 (2)	0.03072 (12)	
O1	0.58250 (8)	0.6496 (2)	0.57328 (6)	0.0249 (2)	
H1O	0.5830 (17)	0.572 (4)	0.5245 (14)	0.055 (6)*	
N1	0.58120 (12)	1.1196 (3)	0.64451 (9)	0.0292 (3)	
H1NA	0.5690 (15)	1.042 (4)	0.5962 (12)	0.037 (5)*	
H1NB	0.6301 (17)	1.238 (4)	0.6523 (12)	0.045 (6)*	
N2	0.47023 (9)	0.6201 (2)	0.56650 (7)	0.0198 (3)	
C1	0.59550 (11)	0.9785 (3)	0.71326 (9)	0.0203 (3)	
C2	0.66473 (11)	1.0555 (3)	0.79012 (9)	0.0235 (3)	
H2	0.705557	1.200830	0.794290	0.028*	
C3	0.67496 (11)	0.9256 (3)	0.85971 (9)	0.0223 (3)	
H3	0.720922	0.983038	0.911337	0.027*	
C4	0.61783 (11)	0.7109 (3)	0.85389 (8)	0.0197 (3)	
C5	0.54751 (10)	0.6311 (3)	0.77926 (8)	0.0172 (3)	
H5	0.507391	0.485190	0.776017	0.021*	
C6	0.53533 (10)	0.7638 (3)	0.70891 (8)	0.0165 (3)	
C7	0.45154 (10)	0.6753 (2)	0.63216 (8)	0.0165 (3)	
C8	0.33591 (10)	0.6438 (2)	0.63101 (7)	0.0156 (3)	
C9	0.29101 (11)	0.8182 (3)	0.66846 (8)	0.0189 (3)	
H9	0.335250	0.950014	0.695888	0.023*	
C10	0.18202 (11)	0.8003 (3)	0.66587 (9)	0.0226 (3)	
H10	0.151395	0.920969	0.690712	0.027*	
C11	0.11782 (11)	0.6062 (3)	0.62701 (9)	0.0229 (3)	
H11	0.043042	0.594435	0.624967	0.028*	
C12	0.16249 (12)	0.4292 (3)	0.59117 (9)	0.0226 (3)	
H12	0.118668	0.294962	0.565375	0.027*	
C13	0.27141 (11)	0.4477 (3)	0.59285 (8)	0.0189 (3)	
H13	0.301768	0.326585	0.567964	0.023*	

### Atomic displacement parameters (Å^2^)

**Table:**

	*U*^11^	*U*^22^	*U*^33^	*U*^12^	*U*^13^	*U*^23^
Cl1	0.0381 (2)	0.0335 (2)	0.01636 (17)	−0.00308 (17)	0.00354 (14)	0.00155 (15)
O1	0.0139 (5)	0.0399 (7)	0.0236 (5)	−0.0007 (4)	0.0097 (4)	−0.0040 (5)
N1	0.0323 (7)	0.0244 (7)	0.0325 (7)	−0.0064 (6)	0.0128 (6)	0.0057 (6)
N2	0.0127 (5)	0.0282 (7)	0.0205 (6)	0.0008 (5)	0.0081 (4)	0.0002 (5)
C1	0.0172 (6)	0.0191 (7)	0.0269 (7)	0.0009 (5)	0.0105 (5)	0.0007 (6)
C2	0.0178 (6)	0.0199 (7)	0.0342 (8)	−0.0042 (6)	0.0107 (6)	−0.0058 (6)
C3	0.0149 (6)	0.0262 (8)	0.0247 (7)	−0.0012 (5)	0.0051 (5)	−0.0081 (6)
C4	0.0177 (6)	0.0235 (7)	0.0176 (6)	0.0017 (5)	0.0057 (5)	−0.0005 (5)
C5	0.0142 (6)	0.0178 (7)	0.0200 (6)	−0.0010 (5)	0.0063 (5)	−0.0023 (5)
C6	0.0132 (6)	0.0182 (7)	0.0192 (6)	0.0009 (5)	0.0069 (5)	−0.0019 (5)
C7	0.0158 (6)	0.0167 (7)	0.0172 (6)	0.0018 (5)	0.0060 (5)	0.0020 (5)
C8	0.0153 (6)	0.0188 (7)	0.0135 (6)	0.0016 (5)	0.0057 (5)	0.0029 (5)
C9	0.0194 (6)	0.0191 (7)	0.0191 (6)	−0.0007 (5)	0.0077 (5)	−0.0010 (5)
C10	0.0209 (7)	0.0246 (8)	0.0260 (7)	0.0029 (6)	0.0128 (6)	−0.0001 (6)
C11	0.0158 (6)	0.0295 (8)	0.0248 (7)	−0.0003 (6)	0.0086 (5)	0.0053 (6)
C12	0.0211 (7)	0.0240 (8)	0.0216 (7)	−0.0059 (6)	0.0055 (5)	0.0002 (6)
C13	0.0207 (6)	0.0197 (7)	0.0171 (6)	0.0003 (5)	0.0073 (5)	0.0001 (5)

### Geometric parameters (Å, º)

**Table:**

Cl1—C4	1.7409 (14)	C5—H5	0.9500
O1—N2	1.4145 (14)	C6—C7	1.4903 (18)
O1—H1O	0.95 (2)	C7—C8	1.4869 (17)
N1—C1	1.3894 (19)	C8—C13	1.3927 (19)
N1—H1NA	0.91 (2)	C8—C9	1.3937 (19)
N1—H1NB	0.89 (2)	C9—C10	1.3871 (18)
N2—C7	1.2809 (17)	C9—H9	0.9500
C1—C2	1.402 (2)	C10—C11	1.386 (2)
C1—C6	1.4065 (19)	C10—H10	0.9500
C2—C3	1.377 (2)	C11—C12	1.385 (2)
C2—H2	0.9500	C11—H11	0.9500
C3—C4	1.383 (2)	C12—C13	1.3913 (19)
C3—H3	0.9500	C12—H12	0.9500
C4—C5	1.3834 (18)	C13—H13	0.9500
C5—C6	1.3920 (19)		
			
N2—O1—H1O	100.5 (13)	C1—C6—C7	123.03 (12)
C1—N1—H1NA	117.7 (13)	N2—C7—C8	116.31 (12)
C1—N1—H1NB	113.8 (13)	N2—C7—C6	125.78 (12)
H1NA—N1—H1NB	112.5 (17)	C8—C7—C6	117.90 (11)
C7—N2—O1	112.76 (11)	C13—C8—C9	119.48 (12)
N1—C1—C2	120.65 (14)	C13—C8—C7	121.94 (12)
N1—C1—C6	121.17 (13)	C9—C8—C7	118.58 (12)
C2—C1—C6	118.03 (13)	C10—C9—C8	120.30 (13)
C3—C2—C1	121.59 (13)	C10—C9—H9	119.9
C3—C2—H2	119.2	C8—C9—H9	119.9
C1—C2—H2	119.2	C11—C10—C9	119.97 (13)
C2—C3—C4	119.53 (13)	C11—C10—H10	120.0
C2—C3—H3	120.2	C9—C10—H10	120.0
C4—C3—H3	120.2	C12—C11—C10	120.11 (13)
C3—C4—C5	120.47 (13)	C12—C11—H11	119.9
C3—C4—Cl1	119.77 (11)	C10—C11—H11	119.9
C5—C4—Cl1	119.76 (11)	C11—C12—C13	120.14 (13)
C4—C5—C6	120.23 (13)	C11—C12—H12	119.9
C4—C5—H5	119.9	C13—C12—H12	119.9
C6—C5—H5	119.9	C12—C13—C8	119.98 (13)
C5—C6—C1	120.10 (12)	C12—C13—H13	120.0
C5—C6—C7	116.75 (12)	C8—C13—H13	120.0
			
N1—C1—C2—C3	176.07 (13)	C1—C6—C7—N2	60.8 (2)
C6—C1—C2—C3	0.5 (2)	C5—C6—C7—C8	55.87 (17)
C1—C2—C3—C4	1.5 (2)	C1—C6—C7—C8	−120.15 (14)
C2—C3—C4—C5	−2.5 (2)	N2—C7—C8—C13	39.36 (18)
C2—C3—C4—Cl1	178.60 (11)	C6—C7—C8—C13	−139.76 (13)
C3—C4—C5—C6	1.4 (2)	N2—C7—C8—C9	−139.76 (13)
Cl1—C4—C5—C6	−179.73 (10)	C6—C7—C8—C9	41.12 (17)
C4—C5—C6—C1	0.7 (2)	C13—C8—C9—C10	−1.7 (2)
C4—C5—C6—C7	−175.41 (12)	C7—C8—C9—C10	177.41 (12)
N1—C1—C6—C5	−177.17 (13)	C8—C9—C10—C11	1.0 (2)
C2—C1—C6—C5	−1.63 (19)	C9—C10—C11—C12	0.4 (2)
N1—C1—C6—C7	−1.3 (2)	C10—C11—C12—C13	−1.1 (2)
C2—C1—C6—C7	174.25 (12)	C11—C12—C13—C8	0.3 (2)
O1—N2—C7—C8	−178.54 (11)	C9—C8—C13—C12	1.05 (19)
O1—N2—C7—C6	0.5 (2)	C7—C8—C13—C12	−178.07 (12)
C5—C6—C7—N2	−123.16 (15)		

### Hydrogen-bond geometry (Å, º)

**Table:**

*D*—H···*A*	*D*—H	H···*A*	*D*···*A*	*D*—H···*A*
N1—H1*NA*···O1	0.91 (2)	2.23 (2)	2.8875 (19)	128.7 (16)
O1—H1*O*···N2^i^	0.95 (2)	1.84 (2)	2.7411 (16)	156.4 (19)

Symmetry code: (i) −*x*+1, −*y*+1, −*z*+1.
